# A missense polymorphism in the putative pheromone receptor gene *VN1R1* is associated with sociosexual behavior

**DOI:** 10.1038/tp.2017.70

**Published:** 2017-04-25

**Authors:** S Henningsson, D Hovey, K Vass, H Walum, K Sandnabba, P Santtila, P Jern, L Westberg

**Affiliations:** 1 Department of Pharmacology, Institute of Neuroscience and Physiology, Sahlgrenska Academy, University of Gothenburg, Gothenburg, Sweden; 2 Department of Medical Epidemiology and Biostatistics, Karolinska Institutet, Stockholm, Sweden; 3 Center for Translational Social Neuroscience, Emory University, Atlanta, GA, USA; 4 Silvio O. Conte Center for Oxytocin and Social Cognition, Center for Translational Social Neuroscience, Atlanta, GA, USA; 5 Yerkes National Primate Research Center, Department of Psychiatry and Behavioral Sciences, Emory University, Atlanta, GA, USA; 6 Department of Psychology, Faculty of Arts, Psychology and Theology, Åbo Akademi University, Åbo, Finland

## Abstract

Pheromones regulate social and reproductive behavior in most mammalian species. These effects are mediated by the vomeronasal and main olfactory systems. Effects of putative pheromones on human neuroendocrine activity, brain activity and attractiveness ratings suggest that humans may communicate via similar chemosignaling. Here we studied two samples of younger and older individuals, respectively, with respect to one nonsynonymous polymorphism in the gene encoding the human vomeronasal type-1 receptor 1, *VN1R1*, and one nonsynonymous polymorphism in the gene encoding the olfactory receptor *OR7D4*. Participants in both samples had self-reported their sociosexual behavior using the sociosexual orientation inventory, including questions regarding lifetime number of one-night stands, number of partners last year and expected number of partners the coming 5 years. In women, there was a significant association between the *VN1R1* polymorphism and sociosexual behavior in both samples, driven specifically by the question regarding one-night stands. Our results support the hypothesis that human social interaction is modulated by communication via chemosignaling.

## Introduction

Pheromones were originally characterized as chemicals that—in contrast to hormones—are secreted outside the body by one individual and detected by another in whom they elicit a behavior or physiological reaction.^1^ In most species—from single-cell organisms to mammals—communication via pheromones is used to signal the sex and social status of an individual and to promote behaviors and endocrine changes essential for mating and aggression.^2, 3, 4, 5^ The question of whether pheromones stimulate social behaviors also in humans remains controversial.

Putative human pheromones include the 16-androstenes androstadien-one (AND), androstenol and androstenone, which are testosterone derivatives present in, for example, human axillary secretions,^6, 7^ and the estrogen derivative estratetraenol.^8, 9^ Effects of exogenous administration of these compounds have been reported for hypothalamus activation,^10, 11, 12^ ratings of (own) mood^13, 14, 15, 16, 17, 18^ and (other's) attractiveness,^19, 20, 21^ and physiological measures,^13, 15, 22, 23^ sometimes in a sex-specific manner.^12, 16, 19, 21, 22, 24^ A component of male sweat has also been shown to affect cycle timing and mood in women.^25^ To show that humans communicate via pheromones in a similar way to other mammals, however, requires an effect on social and reproductive behavior. It has not been decidedly demonstrated that the effects of putative pheromones translate to behavior, but one study provided evidence for an effect of androstenol on behavior such that women exposed to androstenol overnight interacted to a higher degree with men the morning after.^26^ Studies using a secret mixture of putative pheromones as an additive to perfume have suggested effects on sociosexual behavior such that men with additional male pheromones and women with additional female pheromones report engaging in more sexual activities,^27, 28, 29^ although see also ref. 30. Notably, an effect of exogenous administration on behavior does not necessarily imply that humans use this mode of communication via chemosignaling in everyday life or that naturally occurring variation in endogenous levels or function is important for behavioral differences.^12, 22, 31^

The mechanism by which putative pheromones act in humans remains unknown. Pheromone signaling in other mammals was long believed to be mediated solely by the vomeronasal organ (VNO) and accessory olfactory bulb, connecting via the vomeronasal amygdala to the hypothalamus, where neuroendocrine and reproductive functions are regulated.^32^ The VNO organ is present in some humans, but it appears to lack sensory neurons and nerve fibers, and there is no evidence of a functional connection between the VNO and the accessory olfactory bulb.^33, 34^ During the last decade, investigations of mice and pigs have shown that the pathway via the main olfactory epithelium (MOE) and main olfactory bulb is necessary for pheromone signaling to function,^35, 36^ and that the VNO is not necessary for some pheromonal effects.^37^ In mice, there is also evidence that the MOE can mediate pheromonal signals to the vomeronasal part of the amygdala.^38, 39^ Likewise, potential pheromonal effects in humans appear to be mediated via the MOE.^40, 41, 42^ The absence of sufficient evidence for a functional VNO and accessory olfactory bulb in humans^33, 34, 43^ is thus not incompatible with the possibility of human pheromonal signaling.

Almost all of the hundreds of vomeronasal receptor genes in the rodent genome are pseudogenes in humans.^44, 45^ Only five (VN1R1–5) have been reported to be expressed in humans, notably not in the VNO but in the MOE, and to respond in a similar way to other olfactory receptors in cell cultures.^46, 47, 48^ One of the genes necessary for the signal transduction pathway downstream of the vomeronasal receptors in rodents (*Trpc2*) is also a pseudogene in humans, further indicating a different mechanistic function for these receptors in humans compared with rodents.^49^ The natural monoterpene myrtenal and the synthetic agonist Hedione have both been found to activate the human vomeronasal type-1 receptor 1 (*VN1R1*).^48, 50^ The latter has sex-differentiated effects on hypothalamus activity, increasing it significantly more in females than males.^48^ The gene *VN1R1* contains two nonsynonymous single-nucleotide polymorphisms (rs61744949; rs28649880), in complete linkage disequilibrium with each other.

The putative pheromones AND and androstenone have been shown to function as agonists on the olfactory receptor *OR7D4* (family 7, subfamily D, member 4), which is expressed selectively in the main olfactory epithelium.^51^ The uncommon haplotype of two functional, nonsynonymous *OR7D4* polymorphisms (rs61729907; rs5020278), in complete linkage disequilibrium with each other, has been associated with a loss of receptor function, as well as with a less intense and less unpleasant perception of the AND and androstenone odors in both men and women.^52, 53^

With the overall goal of elucidating whether humans communicate via chemosignaling, we have, in the current study, genotyped two of these candidate polymorphisms, that is, the *VN1R1* rs28649880 (A229D) and *OR7D4* rs5020278 (T133M) in a large sample of 3676 individuals who had rated their sociosexual behaviors using the behavioral items of the sociosexual orientation inventory (SOI),^54^ including questions regarding lifetime number of one-night stands, number of sexual partners last year, as well as expected number of partners the next coming 5 years. A smaller sample of 1214 subjects served as replication sample. In both the samples, the polymorphism in the gene encoding the putative pheromone receptor, *VN1R1*, was associated with sociosexual behavior in women.

## Materials and methods

### Participants

The study comprised two different samples: one larger sample including primarily young individuals and one smaller replication sample including older individuals.

Sample one included 3676 participants (2145 women), aged 26±4.6 years (age range: 18–49) with genetic and self-reported behavioral data. The participants were a subset of the second collection of the Genetics of Sexuality and Aggression sample, collected in 2006 (ref. 55) and targeting all Finnish-speaking twin pairs residing in Finland and born in 1973–1988 (that is, 18–33 years old at the time of data collection), as well as their siblings of at least 18 years of age. DNA was provided by 4278 participants. Of those with valid genotype data, 3676 had provided answers on the behavioral measures: 1199 monozygotic and 1502 dizygotic twins, 27 twins of undetermined zygosity and 948 siblings of twins. All the participants provided written informed consent in accordance with the Declaration of Helsinki. The study was approved by the Ethics Committee of the Åbo Akademi University in accordance with the Declaration of Helsinki.

For sample two, genetic and self-reported behavioral data were available for a total of 1214 participants (630 women), aged 61±5 years (age range: 54–69 years). The participants were a subset of the SALTY (Screening Across the Lifespan of Twins Younger) sample, a sample of twins born in Sweden in 1943–1958, contacted in 2009–2010.^56, 57^ Out of the 1214 subjects, 256 were monozygotic and 958 dizygotic twins. The SALTY study was approved by the Ethical review board of Stockholm, Sweden. Informed consent was provided by all the participants.

### Questionnaires and scales

The participants of both samples answered questionnaires comprising the behavioral items of the SOI:^54^ (i) number of lifetime one-night stands, (ii) number of sex partners last year and (iii) number of sex partners they forsee they will have in the coming 5 years. In line with previous studies, the behavioral items were summed to create the sociosexual behavior scale.^54, 58, 59, 60^ The researchers did not know the genotype of the subjects they provided with the questionnaires.

Categories were introduced to reduce the long distribution tail of the items. Those who reported zero to 10 partners were ascribed the corresponding number, whereas the other answers were pooled according to: 11–15=11, 16–20=12, 21–25=13, 26–30=14, 31–40=15, 41–50=16, 51–75=17, 76–100=18, 100–500=19. Winsorisation, that is, recoding the higher values down to a max value, was not considered appropriate for the present samples owing to the consequent large number set to the highest value for item (i) particularly.

In the second sample, the variation in the responses to items (ii) (number of partners last year) and (iii) (expected number of partners the coming 5 years) was low: only 11 men (2%) and 4 women (0.7%) provided answers larger than two on item (ii), whereas 16 men (3%) and 7 women (1%) provided answers larger than two for item (iii). The high age of the participants may render these items less relevant owing to the small percentage of their lives that 1 and 5 years constitute, and due to the fact that majority of these older participants were in long-term relationships (85% of the men and 88% of the women). In this replication sample, there were no values larger than 10 for item (ii). Responses larger than 10 for item (iii) (*n*_M_=2, *n*_F_=1) were categorized as above. Subjects who had never had sex were not considered eligible and excluded from the primary analyses (sample 1: *n*_M_=187 (12%) *n*_F_=208 (10%); sample 2: *n*_M_=4, *n*_F_=2).

Subsequent *post hoc* analyses for the first sample included questions regarding relationship status, duration of current relationship (as indicated by six categories ranging from 1 month to more than 6 years) and sex-related anxiety as determined by the sexual distress scale,^61^ as we judged it possible that these factors could explain variation in sociosexual behavior and/or explain potential associations between olfactory polymorphisms and sociosexual behavior.

For the second sample, *post hoc* variables included relationship status, duration of current relationship, as well as number of lifetime sex partners and number of lifetime romantic relationships. The latter two items were categorized for values larger than 10 in the same manner as the SOI items above in order to reduce the tail of the distribution. These items were not available for sample one, and sex-related anxiety was not available for the replication sample.

### Genotyping

Oragene DNA self-collection kits (DNA Genotek, Ottawa, ON, Canada) were used when collecting saliva samples from the participants. The single-nucleotide polymorphisms were genotyped with KASPar, a competitive allele-specific polymerase chain reaction single-nucleotide polymorphism genotyping system using FRET quencher cassette oligos (LGC Genomics, Hoddesdon, Herts, UK; http://www.lgcgenomics.com).

### Statistical analyses

The website http://www.had2know.com/academics/hardy-weinberg-equilibrium-calculator-2-alleles.html was used to determine, with chi-squared tests, whether the genotype distributions differed significantly from those expected under Hardy–Weinberg equilibrium (HWE), and thus suggested (in the absence of genotyping error) non-random mating or selection effects. The generalized estimating equations procedure in SPSS (version 23, IBM, Armonk, NY, USA) was used to assess the relationship between the SOI behavior variable (dependent variable) and polymorphisms (independent variable). This procedure appropriately controls for dependence arising from genetic relatedness between family members. A linear model was fitted to the data, with an unstructured working correlation matrix as the samples included subjects with different degree of dependence (monozygotic and dizygotic twins). However, as the data were not normally distributed (as ascertained with the Kolmogorov–Smirnov test) but positively skewed and had the nature of count data for time intervals, a negative binomial model was also fitted for the main finding using the same generalized estimating equations procedure and correction family members.^62^ For sample one, the statistical threshold was set to 0.012 (0.05 divided by 4) to control for testing of two polymorphisms and two sexes. Additive (indicated by the subscript 'add' for the *P*-values) models were used, assuming an intermediate phenotypic value for the heterozygote. In addition, recessive and dominant models were examined for the replication sample. *Post hoc* analyses included the three specific variables included in the SOI behavior scale, as well as analyses controlling for relationship status, relationship duration and sex-related anxiety, and number of lifetime sex partners and number of lifetime romantic relationships, for sample one and two, respectively. The fitted models were linear for continuous dependent variables and binary logistic for dichotomous dependent variables. The Wald *χ*^2^ is provided for the generalized estimating equations (as Wald(df)). As this method does not provide estimates of effect size that are easily comparable with other studies, *R*^2^ values or odds ratios, as determined by linear or logistic regression, respectively, are also provided. The power was estimated using 1000 simulated linear regression models and an effect size of *R*^2^=0.005 using the software R.

## Results

### Sample one

The frequency of the uncommon D-allele of *VN1R1* rs28649880 (A229D) was 37% and that of the uncommon M-allele of *OR7D4* rs5020278 (T133M) was 23%. The *OR7D4* was in HWE (*P*=0.6), whereas the *VN1R1* genotype deviated from HWE (*P*=0.0004) by displaying fewer heterozygotes than expected. The lack of HWE for the *VN1R1* polymorphism prompted us to re-genotype this sample with a different KASPar assay and also to genotype the rs61744949, in full linkage disequilibrium with the rs28649880. All monozygotic twins in the sample had the same genotypes as their twin using both assays. The results from the re-genotyping did not indicate any genotyping errors. Exploring the lack of HWE, we found a difference in genotype distribution between women who had had sex (included in the study) and those that had not (see 'Questionnaires and scales' in the 'Materials and methods' section), such that the number of heterozygotes was even lower for those who had never had sex (*P*=0.025, Pearson Chi-square=7.4), an effect that was not dependent on age (*P*=0.16).

**Figure 1:**
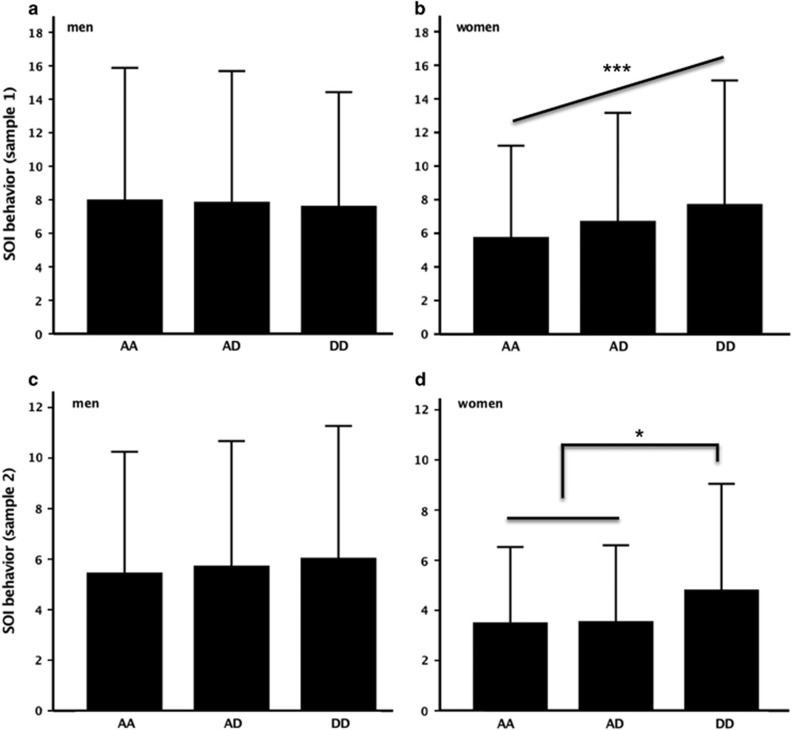
Sociosexual behavior (mean±s.d.) for the different *VN1R1* rs28649880 (A229D) genotypes for (**a**) men in sample one (*P*=NS, *n*=540/618/186), (**b**) women in sample one (*P*_add_<0.0001 for an additive model as determined by generalized estimating equations treating the independent variable representing the three genotypes as a covariate; *n*=813/832/292), (**c**) men in sample two (*P*=NS, *n*=291/213/49) and (**d**) women in sample two (*P*_rec_=0.04 for a recessive model as determined by an independent *t*-test comparing carriers of two uncommon alleles with carriers of at least one common allele; *n*=316/245/42). *P<0.05, ***P<0.001. NS, not significant; SOI, sociosexual orientation inventory.

Sociosexual behavior was significantly associated with the *VN1R1* polymorphism in women (linear model: *P*_add_=0.0001, Wald(1)=14.6, *R*^2^=0.012, negative binomial model: *P*_add_=0.00009, Wald(1)=15.4, Figure 1b), but not men (*P*_add_=0.5, Figure 1a). The gene by sex interaction was significant (*P*_add_=0.001, Wald(1)=11.2). Carriers of the D-allele reported higher sociosexual behavior. Similar results were acquired when we performed the same test for the raw SOI values in women (*P*_add_=0.00008, Wald(1)=15.5, *R*^2^=0.012).

*Post hoc* analyses showed that, in women, the association was strongest for number of one-night stands (item (i): *P*_add_=0.0004, Wald(1)=12.6, *R*^2^=0.009). It was also significant for number of partners last year (item (ii): *P*_add_=0.011, Wald(1)=6.3, *R*^2^=0.006) and expected number of partners the coming 5 years (item (iii): *P*_add_=0.008, Wald(1)=7.0, *R*^2^=0.005). No associations were found in men (*P*-values >0.5). The *OR7D4* polymorphism showed no significant associations (*P*-values >0.5). The power of detecting an effect of *R*^2^>0.005 was above 90% for both polymorphisms for men, and above 95% for women.

In women, the *VN1R1* polymorphism was also significantly associated with relationship status, such that carriers of the D-allele were less likely to be in a relationship (*P*_add_=0.008, Wald(1)=7.1, odds ratio=0.8), but not with relationship duration (*P*_add_=0.9) or sex-related anxiety (*P*_add_=0.9). The association with sociosexual behavior was still significant after controlling for relationship status (*P*_VN1R1_=0.001, Wald(1)=10.4; *P*_status_<0.000001, Wald(1)=83.4), relationship duration (*P*_VN1R1_=0.005, Wald(1)=7.9; *P*_duration_<0.000001, Wald(1)=158) and sex-related anxiety (*P*_VN1R1_=0.0001, Wald(1)=14.9; *P*_anxiety_=0.003, Wald(1)=8.7). The association between *VN1R1* and sociosexual behavior was stronger in the subgroup of women who were in relationships that had endured less than 4 years (*P*_add_=0.01, Wald(1)=6.4, *R*^2^=0.01, *n*=745) than longer (*P*_add_=0.2, Wald(1)=1.7, *R*^2^=0.004, *n*=863). The variation in sociosexual behavior was also larger in those who were in shorter (s.d.: 6.5) than in those who were in longer (s.d.: 3.7) relationships.

### Replication, sample two

The frequency of the uncommon D-allele of the *VN1R1* polymorphism was 28% and the genotype distribution did not differ from HWE (*P*=0.8).

In sample two, female sociosexual behavior was associated with the *VN1R1* polymorphism, but only when assuming a recessive model (linear model: *P*_add_=0.10, *P*_rec_=0.043, Wald(1)=4.1, *R*^2^=0.012, negative binomial model: *P*_rec_=0.021, Wald(1)=5.3, Figure 1d). The corresponding result for the raw values was similar (*P*_rec_=0.046). *Post hoc* analyses showed that the number of one-night stands showed a nominally significant association (*P*_rec_=0.048, Wald(1)=3.9, *R*^2^=0.011), whereas there was no significant relationship between the *VN1R1* polymorphism and item (ii) or (iii) (*P*-values >0.5). The power of detecting an effect of *R*^2^>0.005 was approximately 60% for both polymorphisms.

The association between *VN1R1* and sociosexual behavior was stronger in the subgroup of women who were in relationships that had endured less than 4 years (*P*_rec_=0.018, Wald(1)=5.6, *n*=116) than longer (*P*_rec_=0.3, Wald(1)=1.1, *n*=487), and the relationship between sociosexual behavior and *VN1R1* was no longer significant when controlling for relationship duration (*P*_VN1R1_=0.16, Wald(1)=2.0; *P*_duration_=0.00003, Wald(1)=17.8). The variation in sociosexual behavior was also larger in women who were in shorter (s.d.: 4.7) than in women who were in longer (s.d.: 2.7) relationships. Controlling for relationship status did not change the results markedly (*P*_VN1R1_=0.03, Wald(1)=4.6; *P*_status_=0.02, Wald(1)=5.9). The *VN1R1* polymorphism was not associated with relationship status (*P*_add_=0.9, *P*_rec_=0.8) or with duration of current relationship (*P*_add_=0.6, *P*_rec_=0.2), nor with number of lifetime relationships (*P*_add_=0.7, *P*_rec_=0.4) or number of lifetime sex partners (*P*_add_=0.1, *P*_rec_=0.06).

## Discussion

We have demonstrated an association and a tentative replication of an association between the D-allele of the *VN1R1* rs28649880 A229D polymorphism and higher scores on a self-report measure of sociosexual behavior in women. No association was observed in men and the *OR7D4* polymorphism displayed no significant associations.

The two nonsynonymous polymorphisms in *VN1R1* encode amino acid substitutions in a transmembrane domain (S201F) and in one of the intracellular loops (A229D) of the GPCR, the functions of which remain unknown. Both receptor versions (S-A and F-D) have been shown to be activated by the synthetic VN1R1 agonist Hedione.^48^ Investigations of this ligand indicate that the VN1R1 receptor mediates effects on amygdala activity in both men and women and on hypothalamus activity specifically in women.^48^ Regarding amygdala activity, face-elicited activity has been shown to correlate positively with an increase in sexual partners in young women, whereas the relationship was negative in men.^63^ The sex difference may be relevant to explain that we observed an association between the *VN1R1* polymorphism and sociosexual behavior in women only (Figure 1). Although the female sample was larger, these results did not appear to be due to power differences, as evidenced by similar power estimates for men and women. It is worth noting that the effect sizes were very small explaining only 1% of the variation in female sociosexual behavior in the larger sample.

Carriers of the D-allele in the first sample of younger women were less likely to be in a relationship, and, in both samples, the association between the D-allele and sociosexual behavior was stronger in women who were in shorter (<4 years) relationships. The low number of subjects with shorter relationships in the sample of older women may, therefore, explain why the association was weaker in this sample. As evidenced by the strong correlation, women in shorter relationships generally scored higher on the SOI behavior scale. The variation in sociosexual behavior was also larger in women with shorter relationships, possibly indicating that the power was stronger for those with shorter relationship. In the smaller sample, the association between the D-allele and sociosexual behavior did not remain significant when controlling for relationship duration. This was probably due to diminished power. As there was no association between the *VN1R1* polymorphism and relationship duration, the reported association between the D-allele and sociosexual behavior was probably not a consequence of an influence of relationship duration on sociosexual behavior.

The minor allele frequency for the *VN1R1* polymorphism was larger (37%) in the first sample than in the second (28%) and than has been previously reported,^64^ indicating genetic differences between the samples. Notably, the first sample consisted of younger Finnish people and the second of older Swedes. Population stratification effects were therefore expected.^65^

The genotype distribution in the first sample was not in HWE. This was due to a low number of heterozygotes and not to allele frequencies. As re-genotyping examinations made genotyping error unlikely, we investigated this further, and found that women who had never had sex displayed a significantly more skewed genotype distribution, an effect that was independent of age. A lack of HWE means that the proportions of genotypes in the population differ from those expected if partner choice is random; they differ from those expected if individuals did not have a mating preference for certain genotypes. The lack of HWE in the sample one population may therefore be an indirect indication of the involvement of chemosignaling via VN1R1 in partner preference, though this was not supported by the HWE observed in the second sample. In other mammals, vomeronasal receptor pathways have been implicated in sexual behavior,^66^ sexual motivation^67^ and partner preference.^68^ We speculate that the finding of a *VN1R1* genotype distribution (in the large sample) that deviated from HWE to a higher extent in women who had not had sex, may support similar mechanisms in humans. A recent study supported genetic influences on partner choice in humans by showing a relationship between genetic dissimilarity between partners on the human leukocyte antigen locus and satisfaction with the odor and the sex life with their partner.^69^ Future studies should investigate whether the VN1R1 receptor appears to be involved in partner choice or partner compatibility in humans.

The uncommon W-M *OR7D4* haplotype—comprising the studied rs5020278 polymorphism, encoding an amino acid substitution T133M, and the rs61729907, encoding the substitution R88W—has been associated with a less intense and less unpleasant olfactory perception of AND and androstenone in men and women.^52^ The rs5020278 did not show any significant association with self-reported sociosexual behavior in the current study. We can therefore not shed light on whether potential effects of AND and androstenone on human sociosexual behavior may be mediated by the OR7D4.^12, 13, 26^

Although we cannot rule out that the function of the VN1R1 receptor in the human brain is unrelated to chemosignaling, our results indicate that VN1R1 is a pheromone receptor influencing human sociosexual behavior. As explained in the introduction, the VN1R1 is expressed in human olfactory epithelium,^46^ and due to the lack of evidence for a functional VNO in humans, a VNO-independent mechanism involving the MOE pathway is more plausible.^37, 40, 41, 42^ Substantial evidence suggests that AND and similar molecules are human chemosignals. Their inability to activate VN1R1^48^ argues, however, that other unknown human chemosignals act through VN1R1. Interestingly, unidentified human chemosignals are carried by, for example, sweat and tears^70^ and, at least partly, transferred between subjects by handshaking.^71^ It is worth noting that if pheromone-like compounds have an effect in humans only in virtue of their odors, sex-specific effects would be related to sex-specific odor detection or ratings of intensity or pleasantness of the chemical compounds involved. Neither the putative pheromones AND and androstenone,^52^ nor the VN1R1 agonist Hedione,^48^ do however appear to elicit sex-specific odor perception and some effects of putative pheromones appear to be independent of conscious smell.^31^

The results should be interpreted with caution until replication attempts have succeded in lending support for a role of *VN1R1* variation for olfactory function and/or sociosexual behavior. One of the limitations of this study is the small size of the replication sample and the fact that the genetic structure and age of this sample is different compared with the first sample. Furthermore, the behaviors were measured using self-assessment, which may lead to bias due to the participants' incomplete memory of events.

In conclusion, the reported association suggests that, as a result of genetic variation, naturally occurring endogenous modulation of VN1R1 function affects women's sociosexual behavior, and that humans thus communicate via chemosignaling.
